# Quantitative Modelling in Stem Cell Biology and Beyond: How to Make Best Use of It

**DOI:** 10.1007/s40778-023-00230-7

**Published:** 2023-12-11

**Authors:** Philip Greulich

**Affiliations:** 1 School of Mathematical Sciences, University of Southampton, Southampton, UK; 2 Institute for Life Sciences, University of Southampton, Southampton, UK

**Keywords:** Quantitative modelling, Advanced hypothesis testing, Universality, Over-fitting

## Abstract

**Purpose of Review:**

This article gives a broad overview of quantitative modelling approaches in biology and provides guidance on how to employ them to boost stem cell research, by helping to answer biological questions and to predict the outcome of biological processes.

**Recent Findings:**

The twenty-first century has seen a steady increase in the proportion of cell biology publications employing mathematical modelling to aid experimental research. However, quantitative modelling is often used as a rather decorative element to confirm experimental findings, an approach which often yields only marginal added value, and is in many cases scientifically questionable.

**Summary:**

Quantitative modelling can boost biological research in manifold ways, but one has to take some careful considerations before embarking on a modelling campaign, in order to maximise its added value, to avoid pitfalls that may lead to wrong results, and to be aware of its fundamental limitations, imposed by the risks of over-fitting and “universality”.

## Introduction

Quantitative modelling is the use of mathematical or computational means to imitate real-world processes and to predict their outcomes. Historically, it has been used for centuries in the form of fundamental physical laws, such as Newton’s laws, while biological problems have been subject to mathematical modelling since the first half of the twentieth century. An example is the work of Luria and Delbrück in 1943 who employed mathematical models, tested on bacterial population data, to find that mutations occur without selection pressure [1]. Yet, for a long time, quantitative modelling has not been seen as a core ingredient of biological research. Only in the twenty-first century, the use of quantitative modelling has become ubiquitous, and the way how it is being viewed by the experimental biologists community has turned from widespread scepticism to being a staple ingredient in modern biological research papers, in particular, in developmental and stem cell biology [2–5].

While quantitative modelling is employed in a wide range of biological work, researchers may not always be fully aware how to utilise the opportunities it offers in a best way. Modelling is often seen as a way to confirm experimental findings by reproducing them *in silico*, and having a “model” for the sake of it is perceived as a way to boost a paper’s impact. However, when modelling is used as a mostly decorative element for an already accomplished experimental campaign, the actual benefit of it is often marginal and sometimes questionable at all.

When considering whether and how to use quantitative modelling in a biological context, one should ask three questions: (1) is there anything quantitative modelling can do which cannot be done by experimental means and standard statistical inference; i.e. can it provide sufficient added value? (2) Can modelling replace experiments and can thereby cut costs and save staff time? (3) If modelling could provide added value, how do I employ it effectively, to maximise the scientific value of the work? In the following, I wish to give some ideas and guidance on how to approach these questions and how to make best use of computational and mathematical modelling.

When assessing whether quantitative modelling can provide added value to a biological research project, one should first define what its purpose is. The most common purposes are the following:

1. To confirm a hypothesis previously suggested experimentally, by fitting the corresponding model to the experimental data and reproducing it faithfully.
2. To predict the outcome of a biological process, e.g. gene expression, protein folding, or morphogenesis.
3. To answer research questions and test hypotheses, if this cannot be done by plain experimental means and standard statistical inference methods.
4. To study intrinsic properties of models and relationships between different models theoretically.

In addition to those purposes come others which are nowadays implemented in established software and are often not explicitly perceived as “modelling”, like data clustering, inference of gene regulatory networks [6–8], and cell fate trajectory inference [9–12]. While those often seem to be straightforward statistical methods, they actually involve sophisticated models and model fitting, which require certain assumptions to be met and hyperparameters to be tuned.

In the following, I wish to assess under which circumstances quantitative modelling is fit for those purposes and how to maximise its added value.

## Reconfirm an Experimental Finding by Modelling

When an experiment has delivered some interesting results, and a hypothesis has been suggested and tested through standard test statistics, one could be tempted to have a quantitative model representing that hypothesis to reproduce the experimental data. However, the added value of such an approach is questionable. On the one hand, if a hypothesis has been tested experimentally, having in addition a mathematical model of that hypothesis matching the data provides only a marginally improved certainty of this hypothesis. While modelling can be used to increase the certainty of a hypothesis, computing such a certainty requires not only to test the model of the suggested hypothesis itself, but also all other, competing but reasonable hypotheses need to be tested.^1^ Otherwise, the added certainty from testing a mathematical model is undefined. On the other hand, having a fitted model matching the experimental data is by no means a guarantee that this model is correct. In particular, if the model is complex, with many free parameters, the model can easily be *over-fitted*, meaning that the parameters provide too much freedom for the model to be matched too closely to the data, fitting the noise rather than real trends [13]. The result is that a completely wrong model may fit the data. Even if the number of parameters is low, a model which may share some, but not necessarily the biologically crucial features with the correct model, may also fit the data (see discussion of “universality” in the section “Universality: Curse and Opportunity”. In both cases, such an approach not only has marginal benefit but may even yield wrong results, which when published stay in the public domain.

## Predicting the Outcomes of Biological Processes

The second purpose is the prediction of the outcome of a biological process. Undeniably, quantitative models can be very powerful in achieving this, yet certain conditions need to be fulfilled to be successful. Most commonly, supervised machine learning is employed for this. Although often not seen as that, machine learning is also a form of mathematical modelling: for example, in deep learning, mathematical functions that resemble the connections of neurons (*artificial neural networks (ANN)*) are used, which are *trained*—that is, fitted—to data, in order to extrapolate trends from this data and thus to predict outcomes beyond the data regime [14]. Ironically, this usually leads to over-fitting of a “wrong model”, yet in a desired way: most of the time, the system to be predicted is not actually a neural network (for example, when predicting protein folding, such as by AlphaFold [15]), yet due to the vast number of parameters, those neural networks can fit almost any (labelled) data. However, despite over-fitting, machine learning yields excellent predictions—a feat that has not been entirely understood yet.^2^ An additional method to improve the predictive power of ANNs is *regularisation* [16]: when fitting the ANN, large parameter variations are penalised in the evaluation of the fitting accuracy, so that the parameter space is effectively substantially reduced, leading to an excellent predictive power. Hence, the power of an ANN lies in its ability to be fitted to a large range of data, despite not representing the biological process on a model level. However, for machine learning to be predictive, it requires vast amounts of data, which usually only high-throughput assays, like next-generation sequencing, can deliver.

While powerful for predictions, machine learning approaches cannot reveal information about the underlying biology, since the models themselves do not reflect the biology (and are therefore also called a “blackbox” models). Furthermore, the predictions work only under the circumstances under which the training data has been collected; changes of circumstances, such as a change of phenotype from a mutation, or a change of experimental settings, cannot be predicted without new experimental data.

An alternative are *mechanistic models* that reflect directly the underlying real-world processes [17]. Such models describe underlying biological or biochemical processes in a mathematical form, usually either (1) in the form of differential equations, which express the time evolution of relevant biological quantities as their time derivatives on the one side, and explicit terms expressing the change rates of these quantities on the other side, or (2) as stochastic processes, which include some degree of randomness and whose predictions are probabilistic. Instead of providing a unique outcome, they predict a probability distribution of outcomes. The advantage of a mechanistic model, if trained appropriately and once tested to be predictive, is that it allows to test the effect of particular changes of circumstances, that is, changes in parameters, which can be expressed in terms of the model rules more easily than in a blackbox machine learning model. However, it is highly challenging to develop such a model. It must be complex enough to contain all relevant features, both for prediction and for comparability with the biological scenario, but at the same time, its free parameters must be few, to avoid over-fitting, since in such a case—in contrast to machine learning—fitting the wrong model would undermine the model’s purpose (even if it could be rendered predictive by regularisation). Hence, one can usually only use such a model if the majority of parameters are accurately known through measurements. An example is protein folding, where all relevant physico-chemical parameters of amino acid crosslinking and their thermal motion are accurately known. If not all parameters are known a priori, mechanistic models can rarely make quantitatively accurate *de novo* predictions, but are often able to predict certain qualitative behaviours of a system, for example, whether a certain quantity varies smoothly or abruptly (in a step-change manner) under a change of external parameters [18], or  predict characteristic properties of differentiation, based on bifurcation theory [19].

Overall, quantitative modelling—which includes machine learning—has great potential to predict the outcome of biological processes, but there are limitations: machine learning requires vast amounts of data (which, however, can be obtained by high-throughput technologies) and can only make predictions for circumstances under which the data has been collected, while mechanistic modelling—which reflects the underlying biology and allows to predict changes in circumstances—requires less data but much pre-existing knowledge about the relevant parameters.

## Answer Biological Questions Through Quantitative Modelling

**Fig. 1:**
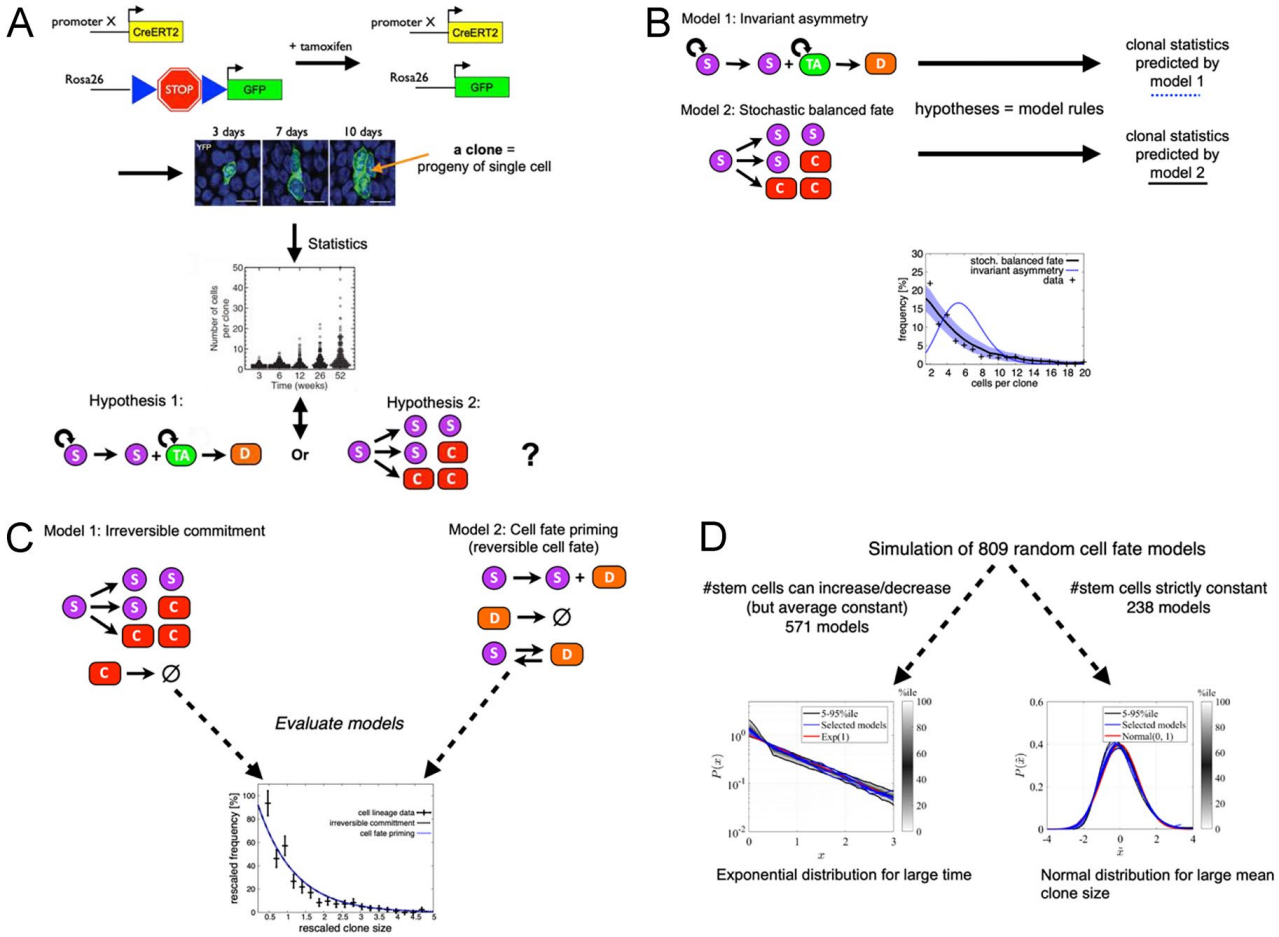
Hypothesis testing via quantitative modelling, exemplified on clonal statistics. **A** Depiction of lineage tracing via Cre-Lox recombination and clonal statistics. Transgenic animals carry a GFP gene preceded by a Stop sequence that is flanked by Lox constructs. The Lox-flanked Stop sequence is removed by a Cre recombinase which is expressed upon administration of tamoxifen, and thus, GFP is expressed. The GFP label is inherited to the progeny of the initial cell, which constitutes a clone and which grows over time upon Cre recombination (centre top, ©2014 SpringerNature. Reprinted, with permission, from [28]). The statistical distributions of clone sizes is recorded (centre bottom, data from P. H. Jones as published in [29]), yet it cannot directly distinguish between hypotheses of cell fate outcome (bottom). **B** Quantitative modelling can bridge the gap between hypotheses and data: each hypothesis represents the rules for a stochastic model of cell fate choice dynamics, which predicts the hypothesis’ expected clonal statistics. The latter can then be directly compared with the experimental data and tested (bottom, data from P. H. Jones, as published in [29]). **C**, **D** Illustration of universality. **C** Two models of stem cell fate choice in homeostasis, which differ in some features, yet predict the same clone size distribution and are thus indistinguishable through static cell lineage tracing data [30] (plot on bottom: ©2019 SpringerNature. Reprinted, with permission, from [31]). **D** ~ 800 randomly generated cell fate models can be categorised in only two universality classes, one predicting an exponential distribution in the long term limit and the other one a normal distribution if mean clone sizes are large (plots reprinted on CC-BY license from [32••]). The two classes are distinguished by only one predictive relevant property, namely, whether the number of stem cells is strictly conserved or not

When attempting to answer a biological question, experimental means and direct hypothesis testing may not be sufficient to yield a clear answer. The experimental data may contain the information relevant to answer the question, but it may not be readily extractable through direct statistical tools. An example is the search for cell fate choice patterns using genetic cell lineage tracing [20•, 21–23] via the Cre-Lox recombinase system [24–26]. The Cre-Lox system allows labelling of cells with fluorescent proteins in an inheritable way, i.e. all of a labelled cell’s progeny caries that fluorescent marker, which allows to trace individual clones (see Fig. 1A). When tissue is harvested and clones analysed, one can obtain the full statistical distribution of clone sizes and their composition of cell (sub-)types if appropriate markers are available. The issue is that, while this statistical distribution is the result of the cell fate choices and thus contains some information about them, it cannot directly reveal the cell fate choice patterns. The problem is twofold: (1) the data is a snapshot of clonal distributions at particular time points, while the biological question is about a *dynamical* process, i.e. the changes of cell type over time and upon cell division, and (2) the data is multicellular, about cell populations (a clone is a sub-population of cells), while the biological question is about the fate of single cells and their daughters upon division. Hence, experimental data and the biological question, that is, any associated hypothesis, do not match in terms of scale of time (static vs. dynamic) and cell number (single- vs. multicellular).

At this point, quantitative modelling can help. It can bridge the gap between biological question/hypotheses and the experimental data, as depicted in Fig. 1B. The key is that every candidate hypothesis can be interpreted as the rules for a mechanistic model, which can be formulated and evaluated mathematically or computationally, as a set of differential equations (deterministic dynamics) or as a stochastic process (which includes random noise), to generate “virtual data”, as would be predicted by that hypothesis. That prediction can then be directly compared with the data and be tested on it.

As an example, let us again consider the search for cell fate choice patterns. Let us assume that we have measured clonal distributions experimentally; yet, we cannot directly see the cell fate choice rules from this data. We can, however, take the possible candidate hypotheses and translate each of them into the update rules of a stochastic process. Then, these models can be evaluated to produce predicted clonal (probability) distributions as output, and the parameters of the models can be fitted by some optimisation method. Those predicted clonal distributions can now be directly compared with the data. Hence, the mathematical modelling turns hypotheses that cannot be compared with the data, into predicted clonal distributions that can be directly overlaid with the data (see Fig. 1B).

Now, if we find that a model’s output cannot be fitted to the data, we can reasonably reject the corresponding hypothesis. However, we have to be careful how to interpret a fitting model output. Can we confirm a hypothesis and settle the question about cell fate choice rules if a predicted clonal distribution fits the data? The answer is a clear “no”; a prediction fitting the data does not confirm a hypothesis, since other candidate hypotheses might as well predict the data equally well or better. And other than being the exception, this rather is the rule. On the one hand, over-fitting, as described before, can allow a wrong model to be fitted to the data if it is formulated with too many free parameters.^3^ Hence, if one does not have the knowledge of all parameters in a possibly very complex biological process, the only reasonable option is to simplify the model so far that the number of parameters is low enough to avoid over-fitting. Thus, many aspects of an initially complex model may need to be neglected (for stochastic models, this simplification means essentially that neglected features are assumed to be random and unbiased, which is covered by a stochastic model’s random noise). On the other hand, often more than one model can fit the data, even if the number of parameters is sufficiently low, since some features that distinguish models may not affect the predictions at all. This phenomenon is often called “universality”, which we discuss in the following section.

### Universality: Curse and Opportunity

*Universality* is the phenomenon that different models can sometimes generate the same predictions with respect to a certain type of data, if some quantities, like mean values or passed time, are sufficiently large [27]. Models which yield the same predictions have some common features, called “predictive relevant”, but may differ substantially in others, called “predictive irrelevant” features (notably, predictive irrelevant features may yet be biologically relevant). Models that differ only in predictive irrelevant features, i.e. yielding the same predictions, can be categorised in one “universality class”, while those that differ in predictive relevant features belong to different universality classes. This has the unfortunate consequence that hypotheses that correspond to models of the same universality class will fit the data equally well and thus cannot be distinguished when the corresponding models are tested against that data. From this also follows that a fitting model does not mean that it is the “correct” model if any of the predictive irrelevant features are biologically relevant for the posed biological question, since any other model of the same universality class, but which may differ in biologically relevant, yet predictive irrelevant features, could fit the data as well.

Universality can have several origins:

- *Weak convergence* [33]: for stochastic processes—which model some degree of randomness—the phenomenon of “weak convergence” means that they generate statistics that converge over time, or if mean numbers are large, to the same limiting distributions, if the predictive relevant features are the same. The most common of these universal limiting distributions is the normal distribution. There is a vast number of random numbers and stochastic processes which all produce a normal distribution and thus are of the same universality class; only few predictive relevant features must be fulfilled for this: (1) the final outcome of the process is a sum of individual steps/random quantities, and (2) the mean value and variance of each step are bounded [34]. Furthermore, the number of steps (interpreted as time steps in a stochastic process) must be large. Notably, any statistical features of individual step sizes, beyond the boundedness of mean and variance, are predictive irrelevant and do not affect outcomes, if the number of steps is large.
- *Non-dimensionalisation*: in both stochastic and deterministic models, quantities and parameters contain physical units, and these units can be arbitrarily chosen. For example, instead of using “seconds” as time unit, one may want to choose the inverse of the cell division rate as time unit, whereby the cell division rate becomes trivially “one division per time unit”. This can be done with other parameters as well, which thus become predictive irrelevant. By *non-dimensionalisation*,^4^ several different models may actually map to the same non-dimensionalised model, with a common prediction, and those thus form the same universality class.
- *Universality of critical phenomena*^5^: complex systems with many interacting components may display critical phenomena, like phase transitions, (e.g. liquid to gas or liquid blood that becomes a solid blood clot). Sufficiently close to the critical points, many models that differ in some features—i.e. the predictive irrelevant features—predict the same functional behaviour of the quantities describing the collective properties of those systems [35, 36]. The predictive relevant features are usually very few and often categorical, for example, which quantities are conserved, what symmetries prevail, and whether the configurations of the system are continuous or discrete (countable in integer numbers).

As an example, consider two cell fate models in homeostasis, as depicted in Fig. 1C. In model 1, a stem cell (S) divides, and upon this division, the daughter cells irreversibly choose their fate, to either remain a stem cell until the next division or to commit to differentiation (C). In model 2, cell divisions are constrained to be always asymmetric, with one cell remaining a stem cell (S) and the other one being primed for differentiation (D), while the cell types may also change independently of cell division, in a reversible way, that is, an S-cell can become a D-cell and a D-cell can reverse to become an S-cell again [30]. Despite these fundamental differences, both models predict the same clone size distribution (Fig. 1C, bottom). Why is this, and what are the predictive relevant features those models share? To answer these questions requires some mathematical analysis, on which we will elaborate later (see also a detailed analysis in Ref. [32••]).

Besides structural features of models, the parameters of a model can be predictive relevant or irrelevant. (Predictive) *Irrelevant parameters* are those which do not change the model predictions at all when changed under conditions where universality prevails; *relevant parameters* are those which affect the model predictions. However, a minimal set of relevant parameters does not necessarily include the plain model parameters; often, predictive relevant parameters are the product or ratio of plain model parameters rather than the parameters themselves. For example, in model 1 of Fig. 1B, the predicted distribution of C-cells depends only on the ratio of division rate and terminal differentiation rate, not explicitly on the individual parameters themselves[20•].^6^ If one has found a best set of parameter values, then doubling both the division rate and the terminal differentiation rate leads to the same best fit, and thus, the “true” best set of parameters is not identifiable [37].

But universality also provides opportunities and thus may be a desired property: if we only wish to distinguish predictive relevant features, and accept for now that we cannot distinguish the predictive irrelevant ones, we do not need to test all models of a universality class but can choose the simplest model—having the lowest number of parameters and being the easiest one to evaluate and analyse—as a representative of that class and thus simplify the whole modelling campaign substantially. Since the predictive features are often categorical, the number of universality classes is usually very small, and thus, only a small number of models, one representative of each universality class, need to be tested. Furthermore, universality is to some extent essential for model testing: models always require some degree of simplification. Universality allows simplifications, that is, negligence of predictive irrelevant features, without compromising the predictive accuracy of a hypothesis/model. Without universality, that is, if all features were predictive relevant, every simplification would lead a model to deviate in its predictions from the data, and even a reasonably “true” model—when subject to some technically necessary simplifications—would not fit the data. This is usually not desired, since simplifications, and be it just for technical reasons, are often essential to evaluate models properly.

Could we overcome the limitations posed by universality? Universality emerges in view of the type of data and the circumstances under which it is collected; other types of data or a change of experimental settings may render certain predictive irrelevant features relevant and thus distinguishable. One could therefore try to obtain richer data with more features. For example, when assessing cell fate choices, one could try to directly observe them through intra-vital live imaging, to gain the time dimension as feature of the data, and with this, further details of the cell fate choices could be distinguished. While such experiments are possible in some circumstances (for example, to observe live cell fate choices in mouse epidermis [38]), they are more expensive in terms of money and effort, more invasive, or not possible in many tissues and situations. On the other hand, universality does not always emerge: usually only in limiting cases, e.g. when experiments are run over longer time scales or when numbers (such as clone sizes) are large, properties are genuinely universal [33]. When data is collected from experiments after shorter time scales or when numbers are smaller, for example, short-term cell lineage tracing after few cell divisions [28, 39], the data, and related model outputs, are not universal, and more features could, in principle, be distinguished. However, this may lead to a trade-off one wishes to avoid: while model details are easier to distinguish for short-term data, reasonable and necessary simplifications to the model may lead to undesired deviations.

To summarise, there is no one-size-fits-all solution, and a lot of intuition is needed to balance the trade-offs between the opportunities and limitations of universality: on the one hand, one wishes to distinguish a sufficient number of features, i.e. having them predictive relevant; on the other hand, one wishes to simplify the models as much as possible, by neglecting predictive irrelevant features. Ideally, the predictive relevant features are the same as the ones relevant to the biological question; this cannot be assured, but appropriate choices of experimental settings can adapt universal features for our purposes, at least to some extent. Unfortunately, the predictive (ir-)relevant features which define the universality classes are often not known beforehand. Then, we may need to travel down the rocky route and follow the classical scientific method, according to K. Popper: come up with a set of all plausible hypotheses and test the corresponding models for all of them; reject those hypotheses which cannot be brought in accordance with the data through fitting, while those that fit (possibly more than one model) may then constitute the universality class of the “true” model. Without prior knowledge about universality classes, however, the number of candidate models to test could be extremely large and arbitrarily complex. Hence, in order to optimise a modelling approach, it is essential to gather some a priori knowledge about the universality classes and their predictive relevant features. This can only be obtained by a mathematical analysis of candidate models’ properties beforehand, as described in the following section.

## Data-Free Model Analysis

Commonly, quantitative models are used in view of experimental data. The study of intrinsic properties of models from a plainly theoretical standpoint, without explicitly taking into account data, is often seen as a mere academic exercise. Nonetheless, studying the intrinsic properties of models, through mathematical and computational tools, can yield impactful insights irrespective of the particular data and can guide and boost experimental campaigns substantially. The crucial benefit of theoretical, data-free model analysis is that it can assess the intrinsic consistency of models and connections between them (that is, between hypotheses), yielding shortcuts for the modelling campaign and guiding experiments. For example, by studying models theoretically, one can:

1. discard some hypotheses a priori, by finding that they are intrinsically not consistent, or are not consistent with the circumstances of experiments.
2. find that some hypotheses imply each other or contradict each other.
3. identify Universality classes and their predictive (ir-)relevant features.

This means that many candidate hypotheses may be redundant, be it because they are intrinsically inconsistent, or follow from/ contradict other hypothesis, or are indistinguishable from others. Needless to say that this may guide towards those experiments that are really needed, and one can identify experiments that are redundant before one starts designing them, thus saving a large amount of effort and costs. An example how hypotheses can be excluded is our study of generic models for tissue cell population dynamics that includes all types of cell fate choice dynamics, in homeostasis [40]. There, it is shown, by introducing rigorous definitions of the concepts of a stem cell type, self-renewal, and homeostasis, that in a homeostatic state, only models where self-renewing cells are at the apex of a lineage hierarchy can prevail; others can be discarded without an expensive model testing campaign. Hence, by using this knowledge, coming from purely theoretical, data-free model analysis, both a lot of modelling and experimental work can be saved. Consequently, data-free model analysis can yield highly valuable information about a biological system that can save a lot of experimental and computational work and thus a lot of money, since the theoretical work is often much cheaper to be done.

Finally, theoretical model analysis can be used to reduce the number of potential candidate hypotheses that need to be tested dramatically, by finding the possible universality classes and identifying the predictive relevant features that associate a model with a universality class. Numerically, this can be done by varying parameters and structural features randomly and test under which circumstances model predictions change and when not. If an appropriate mathematical formulation of the model is available, this can also be done by mathematically taking the limits under which the emergence of universality is suspected (for large times, system size, or close to bifurcations or phase transition points), or taking a coarse-graining process such as “renormalisation” to identify the model behaviour under large scales of time and space [41, 42]. For example, in Ref. [32••], it was shown that in homeostasis, all cell fate models can be categorised in only two universality classes (with further sub-classes when different limits of parameters are considered^7^). The only predictive relevant feature is a binary characteristic: whether the number of cells which retain self-renewal potential is strictly conserved over time, or not (see Fig. 1D). This also explains why model 2 in Fig. 1C, involving cell fate priming and reversibility, predicts the same clone size distribution as model 1: in both cases, the number of stem cells is not conserved, in model 1 via symmetric divisions; in model 2, if upon a division S → S + D, the D-cell turns into an S-cell. Hence, model 2 shares the (only) predictive relevant characteristic with model 1. Another example is the classification of models for cell differentiation via catastrophe theory [19, 43]: many different dynamical systems behave in the same way close to points where the stability of the system changes (“bifurcations”), such as cells when they differentiate. Catastrophe theory was used to show that there are only few possibilities how cells can progress through differentiation [19], which again reduces the number of candidate models to be tested.

## Conclusions

Quantitative modelling can aid biological research in many aspects, but one needs to take into consideration a few things before embarking onto a modelling campaign, in order to make optimal use of it and avoid wrong results. One risk of quantitative modelling, when the exact model is not known a priori and is to be tested on the data, is over-fitting: it is tempting to include a large number of biological details in a model, but this often comes at the cost of additional free parameters, which leads to the phenomenon that an over-fitted, wrong model can seemingly match the data with its output. This can lead to wrong conclusions about the underlying biological hypotheses. Hence, if one wishes to test a model, and thus any underlying hypotheses, the number of free parameters needs to remain low, which requires to keep a model lean, stripping it to the core features that are needed to test the underlying hypothesis.

However, even when a model contains sufficiently few parameters to avoid over-fitting, a matching model might not be the correct one. The reason behind this is “universality” the phenomenon that different models can yield the same predictions, if they possess the same “predictive relevant” features, even if they might differ in other, “predictive irrelevant” features—some of them which may yet be biologically relevant. This means that others than the “true” model may match the data as well. Thus, without prior knowledge about the predictive relevant features of universality classes, a match of model and data does not mean that the model is correct, and the only way to test hypotheses through modelling is by testing *all* reasonable candidate models and exclude those which do not fit.

While the prospect of testing all reasonable models, in conjunction with the rather discouraging fact that many may not be distinguishable at all, seems daunting, the existence of universality classes also provides opportunities, when the universality classes (which are usually very few) and their predictive relevant features are determined beforehand. In that case, only the simplest model, as representatives of each universality class, needs to be tested. If in addition, the features we wish to distinguish biologically are the same as the predictive relevant features, universality is not a problem, yet can simplify the modelling campaign a lot. However, this requires a thorough theoretical, data-free mathematical or computational analysis of the models in question, in order to identify the predictive features and universality classes of relevant models beforehand. In general, data-free theoretical analysis can simplify modelling and experimental research in multiple ways: apart from identifying universal features, one can find criteria to exclude certain models a priori and find connections between models/hypotheses that allow to imply some hypotheses from others, whereby experimental and modelling work can be reduced.

When the aim of modelling is to predict the outcome of a biological process, over-fitting and universality are not problems per se: if one has rich data, e.g. as provided by high-throughput assays, machine learning, together with appropriate tuning of hyperparameters and regularisation, can be used to achieve great predictive power, without necessarily correctly reflecting the underlying biology. However, due to this reason, machine learning does not provide understanding of underlying biological processes and can only make predictions under the conditions where the data was collected. Alternatively, mechanistic models—which directly reflect the underlying biology—can also be used to yield predictions, if almost all parameters are well known beforehand. Such models can in principle be adjusted to make predictions beyond the narrow setup of performed experiments, to perform “in silico” experiments. However, making use of mechanistic models for prediction is very challenging, not only due to the risk of over-fitting but also since, in particular when the situation is not universal (i.e. away from limiting situations), it is difficult or even impossible to find all relevant features. Thus, mechanistic models are rarely used in cell and developmental biology to make de novo predictions but are used in other realms of biology, for example, in epidemiology to predict the spread of infectious diseases [44].

To summarise, mathematical modelling can, when correctly done, provide plenty of added value to biological research. But care needs to be taken when drawing conclusions, since over-fitting or the existence of universality can mislead the model finding process.

## Footnotes

1 The certainty of a hypothesis can be computed as the "Bayesian posterior", but this requires the computation of likelihoods of all reasonable candidate hypotheses.

2 It has been suggested that the training procedure of ANNs exhibits an intrinsic bias towards simple functions that avoid fitting noise and extrapolate predictions well beyond the training data [45, 46].

3 The number of parameters that can be fitted without over-fitting should normally scale with the number of data points, but as described in the “Universality: Curse and Opportunity” section, the phenomenon of “universality” can render parameters irrelevant which means that many instances of those parameters—and not only the “true” parameter values of the underlying real system—can yield a good fit, thus mimicking over-fitting.

4 Since quantities which bear physical units are also called “dimensions”.

5 This is the context in which the term “universality” has been historically introduced and canonically defined. The adaptation of this term to the other situations where predictive irrelevant features exist is non-canonical and not generally used as such in the literature.

6 The reason is that the probability distribution of cell numbers is a Poisson distribution, which is entirely determined by a single parameter, the mean value, which in this case is the cell division rate divided by the terminal differentiation rate.

7 The universality class corresponding to strictly conserved stem cell numbers can yield different predictions in different limits: a normal distribution in the limit of fast stem cell divisions and an exponential distribution for slow stem cell divisions. Thus, this class can be divided into two sub-universality classes.
